# Emerging Roles of Astrocytes in Neuro-Vascular Unit and the Tripartite Synapse With Emphasis on Reactive Gliosis in the Context of Alzheimer’s Disease

**DOI:** 10.3389/fncel.2018.00193

**Published:** 2018-07-10

**Authors:** Cai-Yun Liu, Yu Yang, Wei-Na Ju, Xu Wang, Hong-Liang Zhang

**Affiliations:** ^1^Department of Neurology and Neuroscience Center, The First Hospital of Jilin University, Changchun, China; ^2^Department of Life Sciences, The National Natural Science Foundation of China, Beijing, China

**Keywords:** astrocytes, Alzheimer’s disease, neuro-degeneration, neuro-vascular unit, synapse

## Abstract

Astrocytes, which are five-fold more numerous than neurons in the central nervous system (CNS), are traditionally viewed to provide simple structural and nutritional supports for neurons and to participate in the composition of the blood brain barrier (BBB). In recent years, the active roles of astrocytes in regulating cerebral blood flow (CBF) and in maintaining the homeostasis of the tripartite synapse have attracted increasing attention. More importantly, astrocytes have been associated with the pathogenesis of Alzheimer’s disease (AD), a major cause of dementia in the elderly. Although microglia-induced inflammation is considered important in the development and progression of AD, inflammation attributable to astrogliosis may also play crucial roles. A1 reactive astrocytes induced by inflammatory stimuli might be harmful by up-regulating several classical complement cascade genes thereby leading to chronic inflammation, while A2 induced by ischemia might be protective by up-regulating several neurotrophic factors. Here we provide a concise review of the emerging roles of astrocytes in the homeostasis maintenance of the neuro-vascular unit (NVU) and the tripartite synapse with emphasis on reactive astrogliosis in the context of AD, so as to pave the way for further research in this area, and to search for potential therapeutic targets of AD.

## Introduction

Alzheimer’s disease (AD) is the leading cause of dementia in the aged population. Clinically, AD is a chronic neuro-degenerative disorder characterized by progressive decline of cognitive functions; the decline usually begins with a slight dysfunction of episodic memory, followed by a general dysfunction of overall cognitive abilities, which can affect the life quality of individuals and bring a heavy financial burden to our society (Osborn et al., 2016).

Although AD is generally considered to be a neuronal disease, dysfunctions of the complex interaction between various cell types in the brain are responsible for the pathogenesis of this disease. Astrocytes show a decreased expression of neuronal support and signaling genes, suggesting that astrocytes have the potential to contribute to neuronal dysfunction and cognitive decline in AD (Orre et al., 2014). Here we summarize the roles of astrocytes in the healthy central nervous system (CNS) and in the pathogenesis of AD.

## Astrocyte Biology

Neuroglia was first proposed by Virchow in 1858, which was thought to act as a kind of connective tissue in the brain and be made up of several cell types (Virchow, 1858). In 1893, the term “astrocyte,” vividly describing the stellate morphology of the cells, was first proposed by Lenhossek (1893). We will first review the identification and classification of astrocytes, and their emerging roles in the neuro-vascular unit (NVU) and tripartite synapses.

### Identification

There has been much debate over the identification of astrocytes (Barres, 2003, 2008; Kimelberg, 2004a,b, 2010). Among others, Kimelberg (2010) criteria are more accepted: non-excitability and a negative membrane potential determined by the trans-membrane potassium (K^+^) gradient are necessary but not sufficient for astrocyte identification; glycogen granule, intermediate filament bundles, γ-aminobutyric acid (GABA) and glutamate uptake by astrocyte-specific transporters, processes surrounding blood vessels and synapses and gap junctions between cells, which consist of connexins 30 and 43 are all astrocyte-specific, but not absolute.

Glial fibrillary acidic protein (GFAP) is the major intermediate filament protein in astrocytes (Liem and Messing, 2009). Up-regulation of GFAP expression is known as a sensitive and reliable marker of reactive astrocytes (Sofroniew, 2009). However, it is not a sensitive marker for astrocytes since many mature astrocytes in healthy tissue or remote from lesions express undetectable levels of GFAP (Sofroniew, 2009).

Of note is that GFAP is not exclusive to astrocytes. In the CNS, numerous cells, such as Bergmann glia of the cerebellum, tanycytes at the base of the third ventricle, pituicytes in the neuro-hypophysis, cribrosocytes at the optic nerve head and Müller glia in the retina, can also express GFAP (Sofroniew and Vinters, 2010). These different cell types are generally considered as part of an extended astroglial family (Sofroniew and Vinters, 2010). Besides, pyramidal neurons of the hippocampus of AD patients may also express GFAPΔ135, GFAPΔ164 and GFAPΔEx6, which can be observed in aged controls and Down syndrome patients as well (Hol et al., 2003).

### Classification of Astrocytes

As per differences in the anatomical locations and cellular morphologies, astrocytes are usually classified into two main morphological groups: the protoplasmic astrocytes throughout all the gray matter and the fibrous astrocytes throughout all the white matter (Oberheim et al., 2012). However, the diversity and complexity of cortical astrocytes contribute to one of the most distinguishing characteristics of the adult human brain (Oberheim et al., 2006). Oberheim et al. (2006, 2009) found another two distinct morphologies within the human cortex, i.e., inter-laminar astrocytes in superficial cortical layers, specific for primates and polarized astrocytes in the deep cortical layers.

### Astrocytes in the Neuro-Vascular Unit (NVU)

**Figure 1:**
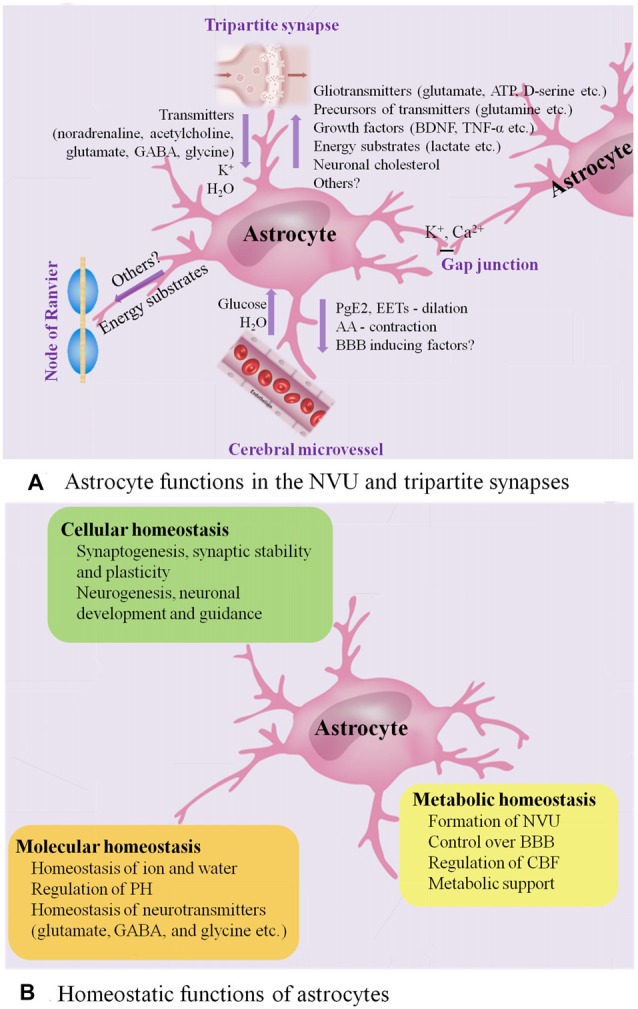
Astrocyte functions in healthy CNS. **(A)** Astrocyte functions in the NVU and tripartite synapses. In the NVU, astrocytes gain metabolic substrates such as glucose and water from cerebral microvessel, while they can release PGE2 and epoxyeicosatrienoic acids (EETs) to evoke vasodilation and blood flow increase and release arachidonic acid (AA) to induce vasocontraction. Close apposition of astrocytic end-feet with the vasculature is vital for BBB. In the tripartite synapses, astrocytes express ionotropic and metabotropic membrane receptors, which can be activated by neuro-transmitters (such as noradrenaline, acetylcholine and glutamate) released from the pre-synaptic cleft, allowing them to sense the intensity of synaptic activity and modulate synaptic function in turn. Astrocytes participate in the spatial regulation of extracellular K^+^ to control the ionic environment of neuropil, another way to modulate neuronal signaling. Ca^2+^-dependent release of gliotransmitters (such as glutamate, ATP and D-serine) allows astrocytes to control the synaptic activity. Astrocyte processes are rich in transporters for neuro-transmitters, including glutamate, GABA and glycine, serving to clear the neuro-transmitters from the synaptic space. Then, the neuro-transmitters taken into astrocytes are converted by enzymes into precursors such as glutamine, recycling back to synapses and reconverting into active transmitters. In addition, astrocytes have the potential to impose powerful and long-term impact on synaptic function by releasing growth factors and related molecules, such as brain-derived neurotrophic factor (BDNF) and TNF-α. Astrocytes have emerged as vital players in the production, delivery, storage and utilization of the brain energy. Lactate synthesized by astrocytes is released to the extracellular space, then taken up by neurons and oxidized to provide energy. Moreover, astrocytes are the main producers of neuronal cholesterol, which is an essential component of membranes and the precursor for many vital signaling molecules. They can also provide energy substrates to the nodes of Ranvier. Finally, astrocytes are interconnected into functional networks via gap junctions. **(B)** Homeostatic functions of astrocytes. Astrocytes play multiple essential roles in the maintenance of cellular, molecular and metabolic homeostasis. ATP, adenosine triphosphate; BBB, blood brain barrier; CNS, central nervous system; GABA, γ-aminobutyric acid; NVU, neuro-vascular unit; PGE2, prostaglandin E2; TNF, tumor necrosis factor.

With multiple end-feet providing almost complete coverage of the cerebral micro-vessels (Mathiisen et al., 2010), astrocytes can act as a “bridge,” sensing synaptic activity and coordinating the delivery of oxygen and glucose with the metabolic requirements of nervous tissue (Belanger et al., 2011; Howarth, 2014; Figure 1A presents astrocyte functions in the NVU).

During neuronal activity, in addition to the release of nitric oxide (NO) by neurons, the calcium (Ca^2+^)-dependent release of vasoactive substances by astrocyte end-feet, such as prostaglandin E2 (PGE2) and epoxyeicosatrienoic acids (EETs), results in activation of K^+^ channels in smooth muscle, evoking vasodilation and cerebral blood flow (CBF) increase (Zonta et al., 2003; Takano et al., 2006; Attwell et al., 2010; Belanger et al., 2011). In addition to being metabolized to PGE2 or EETs within the astrocytes, arachidonic acid (AA) can alternatively diffuse to arteriole smooth muscle, and transform into the vasoconstrictor 20-hydroxyeicosatetraenoic acid (Roman, 2002; Mulligan and MacVicar, 2004; Metea and Newman, 2006).

Besides, the tight relationship between astrocytic end-feet and the vasculature is vital for the integrity of the blood brain barrier (BBB) and the homeostasis of ionic and water. Astrocytes are able to up-regulate various BBB features, contributing to tighter tight junctions, the expression and polarized localization of transporters, and specialized enzyme systems, which are physical barrier, transport barrier and metabolic barrier, respectively (Abbott et al., 2006; Lécuyer et al., 2016; Zenaro et al., 2017). Astrocytes play a primary role in the expression of the tight junction proteins, including occludin, claudin-5 and zonula occludens-1 (ZO-1) in the mature brain vasculature, which correlate with the induction and maintenance of the BBB integrity (Willis et al., 2004). Astrocyte membranes are abundant in aquaporin 4 (AQP4) water channels and K^+^ transporters, as well as different kinds of proton shuttling, including mono-carboxylic acid transporters, bicarbonate transporters, the Na^+^/H^+^ exchanger and the vacuolar-type proton adenosine tri-phosphatase (ATPase), all of which play a critical role in regulating ionic, water and potential of hydrogen (PH) homeostasis (Sofroniew and Vinters, 2010). An astrocytic AQP4-dependent anatomical pathway called “glymphatic” system promotes the exchange of interstitial fluid (ISF) and cerebro-spinal fluid (CSF) and the clearance of interstitial solutes, such as amyloid β (Aβ; Iliff et al., 2012).

### Astrocytes in the Tripartite Synapses

Astrocytes, together with presynaptic and postsynaptic nerve terminals, form “tripartite” complexes (Araque et al., 1999). Peri-synaptic processes of astrocytes can modulate the stabilization, dynamics and maturation of dendritic spines, and participate in the modulation of synaptic transmission and plasticity (Ullian et al., 2001; Haber et al., 2006; Nishida and Okabe, 2007; Rossi, 2015; Figure 1A presents astrocyte functions in the tripartite synapses).

Astrocytes express ionotropic and metabotropic membrane receptors, which can be activated by neuro-transmitters (for instance, noradrenaline, acetylcholine and glutamate) released from the pre-synaptic cleft, allowing them to sense the intensity of synaptic activity and modulate synaptic function in turn (Verkhratsky and Nedergaard, 2014). In this process, transient increase of astrocytic intra-cellular Ca^2+^ levels, the degree of which is dependent on the intensity of neuronal activity, triggers the selective release of various glio-transmitters (for instance, adenosine triphosphate (ATP), glutamate and D-serine) to individual synaptic inputs, offering multiple ways of controlling synaptic activity (Dani et al., 1992; Nedergaard, 1994; Parpura et al., 1994; Porter and McCarthy, 1996; Pasti et al., 1997; Araque et al., 1998, 2014; Fellin et al., 2004; Perea and Araque, 2007; Henneberger et al., 2010; Di Castro et al., 2011; Min and Nevian, 2012; Navarrete et al., 2012; Khakh and McCarthy, 2015; Rusakov, 2015).

In addition, spontaneous Ca^2+^ oscillation, intrinsic signaling detected in a subpopulation of astrocytes, is independent of neuronal activity and may also be involved in modulating neuronal activity (Nett et al., 2002). Up-regulation of astrocytic Ca^2+^ has a direct influence on synapses (synaptic integrity and plasticity, the release of glio-transmitters) and circuit transmission (sensory plasticity and network synchronization; Guerra-Gomes et al., 2018). Moreover, astrocytes can remove synaptically released excitatory and inhibitory neuro-transmitters known as glutamate and GABA respectively, the speed of which affects the intensity of post-synaptic activation, serving to further regulate signal transmission (Arnth-Jensen et al., 2002; Schousboe et al., 2004). Astrocytes take part in the spatial regulation of extracellular K^+^ to control the ionic environment of neuropil, another way to modulate neuronal signaling (Halassa and Haydon, 2010). Astrocytes are also capable of imposing powerful and long-term impact on synapses by releasing growth factors and related molecules, including brain-derived neurotrophic factor (BDNF) and tumor necrosis factor-α (TNF-α; Sofroniew and Vinters, 2010).

Astrocytes are inter-connected into functional networks via gap junction channels and simultaneously integrate information from the neurons and beyond the synapses, such as from microglia and vascular cells, modulating the complex neuro-transmission within a dynamic micro-environment finely (Giaume et al., 2010). Interestingly, the transplant-derived human glia progenitor cells were gap-junction-coupled to astrocytes of host mouse and show some human-like physiological properties, for example, propagating Ca^2+^ signals three-fold faster than their hosts resulting in enhanced long-term potentiation (LTP) and memory and learning functions of the transplanted mice (Han et al., 2013).

Astrocytes in the tripartite synapses also play an essential role in the homeostasis of transmitters (Sofroniew and Vinters, 2010). Astrocyte processes are rich in transporters for neuro-transmitters, including glutamate, glycine and GABA, serving to remove the neuro-transmitters from the synaptic cleft (Sofroniew and Vinters, 2010). Then, the neuro-transmitters taken into astrocytes are converted by enzymes into precursors, such as glutamine, recycling back to synapses and reconverting into active transmitters (Sofroniew and Vinters, 2010). In this way, astrocytes also play a protective role by maintaining a sufficiently low level of extra-synaptic glutamate to prevent excitotoxicity (Schousboe et al., 2004), and releasing anti-oxidants such as glutathione to protect neurons from oxidative stress (Chen et al., 2001, 2009; Shih et al., 2003; Vargas et al., 2008).

Astrocytes have emerged as vital players in the production, delivery, storage and utilization of the brain energy including glucose, as well as its metabolic intermediates, such as lactate, ketone bodies, glutamate and pyruvate (Zielke et al., 2009; van Hall et al., 2009; Belanger et al., 2011; Patel et al., 2014; Magistretti and Allaman, 2015; Camandola and Mattson, 2017; Clasadonte et al., 2017; Liu et al., 2017).

Moreover, astrocytes are the main producers of neuronal cholesterol, which is an essential component of membranes and the precursor for many vital signaling molecules (Pfrieger and Ungerer, 2011; Zhang and Liu, 2015).

In summary, astrocytes are highly differentiated cells that play several essential roles in the healthy CNS, including maintenance of the homeostasis of fluid, ions and transmitters, regulation of CBF, participation in synaptic plasticity and functions, and provision of energy substrates to neurons (Figure 1). Increasing evidences indicate that astrocytes participate in regulation of advanced brain functions, including coordinating fine motor (Saab et al., 2012), controlling the sleep-wake cycle (Ding et al., 2016; Brancaccio et al., 2017), regulating memory (Orr et al., 2015), modulating depressive-like behaviors (Cao et al., 2013; Wang et al., 2017; Yang et al., 2018) and influencing ageing rate (Yin et al., 2017). In response to various forms of insults including ischemia, trauma, infections and neuro-degenerative diseases such as AD, astrocytes undergo extensive cellular, molecular and functional changes whereby losing normal functions or gaining detrimental effects. Herein, we will focus on the emerging role of astrocytes in AD.

## Astrocytes in Alzheimer’S Disease

**Figure 2:**
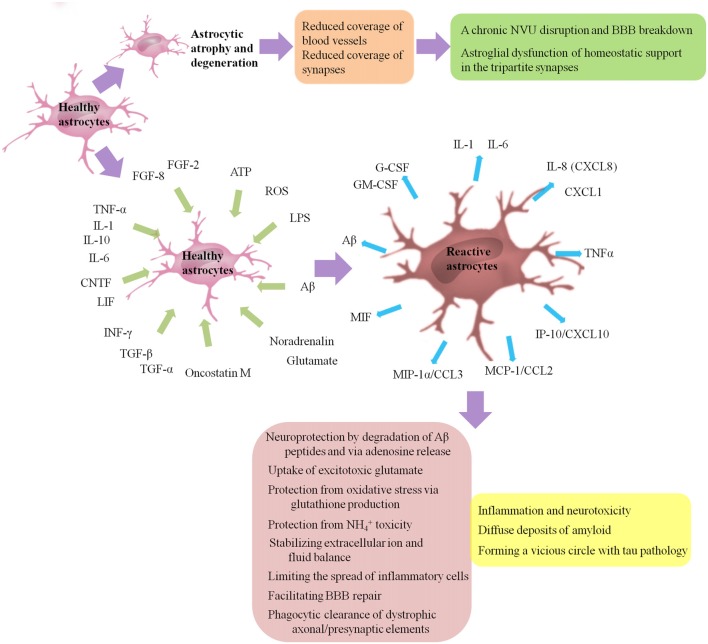
Astrogliosis and astro-degeneration in AD. Astrocytes undergo differential pathological alterations, depending on the stage of the disease, their relation to Aβ plaques and distinct brain regions. Both astrogliosis and astro-degeneration occur in AD, and often precede the formation of specific histopathology. First, at the early stages of AD, astrocytes in EC, prefrontal cortex and hippocampus exhibit features of atrophy and degeneration, reduction in volume of GFAP-positive profiles, surface area and morphological complexity. Astroglial atrophy and degeneration most likely lead to a reduction in the astroglial coverage of blood vessels and synapses, contributing to dysfunction in the NVU and tripartite synapses. Second, astrogliosis is a finely gradated continuum of progressive alterations in gene expression and cellular changes, which may be triggered or regulated by many various intercellular signaling molecules, including IL-1, IL-6, IL-10, TNF-α, INF-γ, CNTF, LIF, oncostatin M, FGF-2, FGF-8, TGF-α, TGF-β, Aβ, LPS, ATP, ROS, noradrenalin and glutamate. Activated astrocytes may secrete many cytokines and chemokines, such as IL-1, IL-6, TNF-α, CXCL1, IL-8 (CXCL8), IP-10/CXCL10, MCP-1/CCL2, MIP-1α/CCL3, MIF, G-CSF and GM-CSF, causing the infiltration of circulating leukocytes into the brain and leading to a chronic inflammatory process. Besides, reactive glial cells are closely associated with diffuse deposits of Aβ. A normal process of astrogliosis exerts beneficial functions, including: neuroprotection by degradation of Aβ peptides and via adenosine release; uptake of excitotoxic glutamate; protection from oxidative stress via glutathione production and protection from NH_4_^+^ toxicity; stabilizing extracellular ion and fluid balance; limiting the spread of inflammatory cells; facilitating BBB repair; phagocytic clearance of dystrophic axonal/presynaptic elements. Pathologically aggregated tau is also closely associated with gliosis. On one hand, tau pathology is capable to promote microglial and astrocytic activations. On the other hand, activation of microglia/astrocytes and associated pro-inflammatory cytokines such as TNF promote tau pathology in turn, forming a vicious circle. Aβ, amyloid-β; AD, Alzheimer’s disease; ATP, adenosine triphosphate; CNTF, cilliary neurotrophic factor; CCL, chemokine C-C motif ligand; CXCL, chemokine C-X-C motif ligand; EC, entorhinal cortex; FGF, fibroblast growth factor; G-CSF, granulocyte colony stimulating factor; GFAP, glial fibrillary acidic protein; GM-CSF, granulocyte-macrophage colony stimulating factors; IL, interleukin; INF-γ, interferon-γ; IP-10, INF-γ-induced protein 10; LIF, leukemia inhibitory factor; LPS, lipopolysaccharide; MCP-1, monocyte chemoattractant protein 1; MIF, macrophage migration inhibitory factor; MIP-1α, macrophage inflammatory protein 1 alpha; ROS, reactive oxygen species; TGF, transforming growth factor; TNF-α, tumor necrosis factor-α.

AD is histo-pathologically characterized by the presence of senile plaques with Aβ aggregates, intracellular neurofibrillary tangles (NFTs) with hyper-phosphorylated tau and activated glial cells surrounding senile plaques (Serrano-Pozo et al., 2013). Both astrogliosis and atrophic/asthenic changes of astrocytes (termed “astro-degeneration”) occur, and often precede the formation of specific histopathology, including plaques and NFTs (Heneka et al., 2005; Verkhratsky et al., 2010, 2014, 2016; Orre et al., 2014; Figure 2 presents astrogliosis and astro-degeneration in AD). We then focus on how astrogliosis and astro-degeneration occur and their roles in AD.

### Astrogliosis

Gliosis, including the activation and often proliferation of glial cells, is a non-specific phenomenon observed in neuro-degenerative diseases and brain injury. There is increasing recognition that reactive astrocytes are vital and central responders to CNS injury and disease, but there is no widely accepted definition of reactive astrogliosis. A definition of astrogliosis encompassing four interdependent key features has been proposed by Sofroniew (2009): (1) astrocytes respond to CNS insults including subtle perturbations and a series of changes occur; (2) a gradated continuum of progressive changes in molecular expression and progressive cellular hypertrophy may occur depending on the intrinsic quality and severity of the insult, and in severe cases, proliferation and scar formation may be initiated; (3) the changes undergone during reactive astrogliosis are regulated by inter- and intra-cellular signaling molecules in a context-specific manner; and (4) surrounding neurons and non-neural cells may be influenced by the altered activities of astrocytes following astrogliosis. Taken together, astrogliosis can be defined as a finely gradated continuum of progressive cellular and molecular changes in astrocytes. Reactive astrocytes exhibit lots of molecular and morphological characteristics. As discussed above, most if not all reactive astrocytes are GFAP positive, while some astrocytes express undetectable levels of GFAP in healthy CNS tissue or remote from lesions. Up-regulation of GFAP, as well as up-regulation of vimentin and synemin, reexpression of nestin and hypertrophy of astrocytes, is viewed as a marker of astrogliosis.

Liddelow et al. (2017) proposed that neuro-inflammation and ischemia induced two distinct types of reactive astrocytes termed “A1” and “A2” respectively. A1s are highly present in many neuro-degenerative diseases, including AD, as well as Parkinson’s disease (PD), Huntington’s disease (HD), amyotrophic lateral sclerosis (ALS) and multiple sclerosis (MS). Several classical complement cascade genes are up-regulated in A1 astrocytes, which have been demonstrated to be destructive to synapses (Stevens et al., 2007). A1 cells lose the ability to promote the survival and outgrowth of neurons and to promote synaptogenesis and to phagocytize debris of synapses and myelin, secrete a still unclear neurotoxin, and finally drive death of neurons and oligodendrocytes. By contrast, A2 cells induced by ischemia have the potential to up-regulate many neurotrophic factors and thrombospondins, which promote survival and growth of neurons and promote synapse repair, respectively.

Many intercellular signaling molecules have the potential to trigger or regulate reactive astrogliosis. A number of cytokines and growth factors (Balasingam et al., 1994; John et al., 2003; Cregg et al., 2014), including interleukin (IL)-1, IL-6 (Klein et al., 1997; Hostenbach et al., 2014), TNF-α (Rabchevsky et al., 1998), interferon (INF)-γ (Yong et al., 1991), leukemia inhibitory factor (LIF), cilliary neurotrophic factor (CNTF; Winter et al., 1995), oncostatin M (Sriram et al., 2004), fibroblast growth factor (FGF)-2, FGF-8 (Kang K. et al., 2014), transforming growth factor (TGF)-α and TGF-β, have been shown able to activate astrocytes. Aβ, as well as mediators of innate immunity such as lipopolysaccharide (LPS), purines such as ATP, neuro-transmitters such as noradrenalin and glutamate, and molecules of oxidative stress such as reactive oxygen species (ROS), also has the potential to regulate astrogliosis (Sofroniew, 2009). There is some debate about whether FGFs promote or suppress the activation of astrocytes (Eclancher et al., 1990; Menon and Landerholm, 1994; Reilly and Kumari, 1996; Reilly et al., 1998; Clarke et al., 2001; Goddard et al., 2002; Kang W. et al., 2014; Kang K. et al., 2014; Hizay et al., 2016), which needs further research.

In response to various forms of CNS insults, such mediators of astrogliosis could be released by any cell types in CNS, such as other astrocytes, oligodendrocyte lineage cells, neurons, microglia, leukocytes, endothelia and pericytes (Sofroniew, 2009). It was recently demonstrated, *in vitro* and *in vivo*, that activated microglia, which were insufficient to kill neurons by themselves, could strongly induce A1s by secreting IL-1α, TNF-α and complement component 1, q subcomponent (C1q), which together were sufficient and necessary (Liddelow et al., 2017). Future studies should focus on the question what the neurotoxin secreted by A1s is, and on new drugs which are potential to treat various CNS diseases including AD by preventing A1 formation, promoting A1 reversion, or blocking the neurotoxin, and on whether available drugs approved to inhibit human IL-1α and TNF-α could be applied on AD. Interestingly, CCR2−/− mice with reduced monocyte invasion show strong increased astrocyte proliferation but reduced scar formation (Frik et al., 2018). Further studies should focus on the signaling pathways underlying the adverse effects of monocyte invasion.

The regulatory mechanisms of astrogliosis remain elusive. Dramatically, transcriptomic analysis shows that gene expression of reactive astrocytes varies depending upon the stimulus (Zamanian et al., 2012). Accordingly, the specific contexts of stimuli induce different morphological, molecular and functional alterations in reactive astrocytes via distinct inter- and intra-cellular signaling pathways, which is relevant to various roles of astrocytes playing in different pathological states (Sofroniew, 2009). The diverse signaling pathways associated with signal transducer and activator of transcription 3 (STAT3), NF-κB, cAMP, Rho-kinase, JNK, Olig2, CEPB1 and EphB2 are involved in mediating different aspects or different degrees of astrogliosis, including cell hypertrophy, proliferation, migration and scar formation as well as up-regulation of GFAP, vimentin and nestin (Sofroniew, 2014). Among these signaling pathways, the Janus kinase (JAK)-STAT3 pathway is a crucial inducer of astrocyte reactivity in various pathological conditions in the CNS (Ceyzeriat et al., 2016). The NF-κB pathway is strongly associated with neuro-inflammation and neuro-degenerative diseases and can be activated by numerous pro-inflammatory agents, such as cytokines, viral or bacterial antigens, Aβ and free radicals (Liddelow and Barres, 2017). In addition to NF-κB activation, complement protein C3 (a marker of A1 astrocytes) can be activated by Aβ in brain tissue from APP transgenic mice and AD patients (Lian et al., 2015). Moreover, it has been shown that β1-integrin-mediated signaling is essential for the procurement of mature, non-reactive astrocytes and, therefore, alterations of this signaling pathway may be implicated in eliciting reactive astrogliosis (Robel et al., 2009). A recent research in mice has shown that active ErbB signaling is able to stimulate many intracellular signals crucial for astrogliosis, including STAT3 and is sufficient to initiate reactive responses of mature astrocytes, prompting characterized morphological and molecular features of injury- and/or disease-induced astrogliosis (Chen et al., 2017).

The overall process of astrogliosis was initially viewed as a maladaptive and negative phenomenon, contributing to inflammation and neurotoxicity. Astrocytes may secrete many cytokines and chemokines, such as IL-1, IL-6, chemokine C-X-C motif ligand (CXCL)-1, IL-8 (CXCL8), INF-γ-induced protein 10 (IP-10/CXCL10), TNF-α, monocyte chemoattractant protein 1 (MCP-1/chemokine C-C motif ligand (CCL2)), macrophage inflammatory protein 1 alpha (MIP-1α/CCL3), macrophage migration inhibitory factor (MIF), granulocyte-macrophage colony stimulating factors (GM-CSF) and granulocyte colony stimulating factors (G-CSF), causing the infiltration of circulating leukocytes into the brain and leading to a chronic inflammatory process, which can be promoted by activated perivascular microglial cells (Liebner et al., 2018). The persistent activation of glial cells and associated inflammation may be part of the neuro-inflammatory neurotoxic response (Wyss-Coray, 2006; Lian et al., 2015; Kawano et al., 2017) and associated with the progression of AD (Nagele et al., 2004; Osborn et al., 2016).

Increasing evidences have shown that a normal process of astrogliosis exerts positive effects, including (Sofroniew and Vinters, 2010; Gomez-Arboledas et al., 2018): (1) neuroprotection by degradation of Aβ peptides and via adenosine release; (2) protection against oxidative stress via producing glutathione and protection from NH_4_^+^ toxicity; (3) excitotoxic glutamate uptake; (4) stabilizing extracellular ion and fluid balance; (5) limiting the spread of inflammatory cells; (6) facilitating BBB repair; and (7) phagocytic clearance of dystrophic axonal/presynaptic elements. A recent study on post-mortem brains obtained from bodies of AD patients aged over 70 years at death showed that reactive astrocytes with enhanced glutamate transporter (GLT)-1 expression may protect neurons and synaptic transmission from the neurotoxicity due to Aβ and NFT deposition, and then help to maintain cognitive function despite the progression of AD neuro-pathological alterations (Kobayashi et al., 2018).

Concerning Aβ, reactive glial cells are strongly related with Aβ plaque formation or diffuse deposits of Aβ (Nagele et al., 2004). Meanwhile, activated microglia and reactive astrocytes surrounding Aβ plaques are also regarded as an endogenous defense mechanism against plaque deposition in AD (Wyss-Coray et al., 2003; Koistinaho et al., 2004; Condello et al., 2015). Pathologically aggregated tau, another histo-pathological hallmark of AD, is also closely associated with gliosis. There is a co-localization between tau oligomers and activated microglia/astrocytes, which has been demonstrated in both tauopathy mice and AD patients (Sasaki et al., 2008; Nilson et al., 2017). On one hand, tau pathology is capable to promote microglial and astrocytic activation. Several studies on tau transgenic mice have demonstrated that significant astrogliosis (Laurent et al., 2017), as well as microgliosis and synapse loss (Bellucci et al., 2004; Yoshiyama et al., 2007), is spatio-temporally associated with hippocampal tau pathology. In the context of tau pathology, T cell infiltration may directly regulate the activation status of microglia and/or astrocytes, exerting passive influence on synaptic plasticity (Laurent et al., 2017). *In vitro*, misfolded truncated tau protein may activate microglia and induce the release of pro-inflammatory cytokines (TNF-α, IL-1β, IL-6) and NO via MAPK pathways (Kovac et al., 2011). On the other hand, activation of microglia/astrocytes and associated pro-inflammatory cytokines such as TNF-α promote tau pathology in turn (Gorlovoy et al., 2009; Maphis et al., 2015; Laurent et al., 2018), forming a vicious circle. Furthermore, *in vitro* (Calafate et al., 2015; Takeda et al., 2015) and *in vivo* (de Calignon et al., 2012; Liu et al., 2012; Kaufman et al., 2017; Narasimhan et al., 2017; DeVos et al., 2018) studies have provided evidence that tau aggregates have the ability to spread along the synaptically connected networks. In the tripartite synapses, tau may contribute to the dysfunction of neurons and synapses due to reduced density of dendritic spines, deterioration of axon initial segments, impairment of axonal transport and reduced mobility of presynaptic vesicles (Tracy and Gan, 2018). Truncated tau may also have the potential to exacerbate BBB deterioration, featured by an increase of mannitol permeability and a decrease of trans-endothelial electrical resistance, which may be regulated by chemokines and pro-inflammatory cytokines released by activated microglia and astrocytes, such as MCP-1 and TNF-α (Kovac et al., 2009). Blair et al. (2015) demonstrated in a tauopathy mouse model that tau aggregation alone was sufficient for BBB damage. Notably, the integrity of the BBB may recover when the levels of perivascular tau are reduced, suggesting that therapies targeting tau can alleviate the vascular involvement of tauopathies by maintaining BBB integrity (Blair et al., 2015).

### Astro-degeneration

Antibodies against GFAP, a component of a cyto-skeleton, as well as antibodies against S100β and glutamine synthetase (GS), two kinds of cytosolic proteins, are commonly used to identify the morphological appearance of astrocytes. In post-mortem tissues of family AD patients, atrophic astrocytes have been found (Rodríguez-Arellano et al., 2016). Meanwhile, studies in the triple transgenic animal model of AD (3xTg-AD) have demonstrated that astrocytes undergo differential pathological alterations, depending on the stage of the disease, their relation to Aβ plaques and distinct brain regions. First, at the early stages of AD, astrocytes in entorhinal cortex (EC), pre-frontal cortex and hippocampus exhibited features of atrophy and degeneration, reduction in the morphological complexity, surface area and volume of GFAP-positive profiles, which was observed in 3xTg-AD, as well as in another model of AD, the PDAPP-J20 trans-genic mice (Beauquis et al., 2013, 2014). In the 3xTg-AD mice, the atrophic changes appeared much later in the hippocampus than in EC, at the age of 6 months and 1 month respectively, while at the age of 3 months these alterations became significant in the prefrontal cortex (Olabarria et al., 2010; Yeh et al., 2011; Kulijewicz-Nawrot et al., 2012). Conversely, astrocytes surrounding plaques showed obviously hypertrophic characteristics, including increased surface and volume of GFAP-positive profiles (Olabarria et al., 2010). Finally, GS expression and the amount of astrocytes expressing GS in the hippocampus and prefrontal cortex were significantly decreased (Olabarria et al., 2011; Kulijewicz-Nawrot et al., 2013), while GS levels remained stable in EC (Yeh et al., 2013). However, the density of astrocytes was impacted neither by age nor by AD (Olabarria et al., 2010). Insulin-like growth factor receptor (IGFR) signaling may be essential to regulate mitochondrial metabolism and Aβ uptake in astrocytes. Age-related astrocytic dysfunction caused by IGFR signaling deficiency may contribute to the pathogenesis of AD, as well as other age-associated cognitive disorders (Logan et al., 2018). Altered astrocytic expression of AQP4 and GLT-1 results in the disruption of water and glutamate homeostasis, which may be associated with the progressive neuro-degeneration in AD (Hoshi et al., 2018). Further studies are needed to explore the mechanism how astro-degeneration occurs and the signaling pathways by which astro-degeneration works in the pathogenesis of AD.

Astroglial atrophy and degeneration most likely lead to a decrease in the astroglial envelope of cerebral vessels and synapses, contributing to dysfunction in the NVU and tripartite synapses. Within the NVU, the reduction of end-feet coverage of cerebral microvessels results in the vascular deficits as observed at the early stages of AD (Montagne et al., 2015, 2017; Zhao et al., 2015; van de Haar et al., 2016a,b; Sweeney et al., 2018). Experimental studies in transgenic mice have shown that aberrant astrocyte-pericyte signaling leads to a chronic NVU disruption and BBB break-down, contributing to accumulation of circulating neurotoxic molecules including hemoglobin, thrombin, fibrinogen, free iron, hemosiderin and/or plasmin in the CNS, particularly in neurons, as well as faulty clearance of neurotoxic molecules from brain, inappropriate delivery of energy metabolite, and abnormal expression of matrix molecules, vascular receptors and growth factors, all of which may initiate and/or eventually result in neuro-degeneration (Zlokovic, 2008; Ryu and McLarnon, 2009; Hultman et al., 2013; Sengillo et al., 2013; Zhao et al., 2015; Nelson et al., 2016). As discussed above, astrocytes play essential roles in the tripartite synapses, including supply of energy, regulation of synaptic plasticity and transmission and maintenance of the homeostasis of transmitters. Then, astro-degeneration may directly contribute to astroglial dysfunction of homeostatic support in the tripartite synapses, leading to dwindling synaptic contacts, weakening synaptic plasticity and affecting synaptic transmission (Coleman et al., 2004).

Taken together, astrogliosis as well as astro-degeneration, alone or in combination, is the essential component of AD. Further studies on the involvement of astrocytes in the pathogenesis of AD, interaction between astrocytes and other cell types in CNS, and the underlying mechanisms are critical for advancing new therapeutic targets.

## Conclusion

Healthy astrocytes play multiple vital roles in maintaining and regulating synaptic physiology, neuronal communication and energy metabolism, whereas astrogliosis and astro-degeneration contribute to the pathogenesis and progression of AD via both loss of these normal functions and gain of several toxic effects. Astrogliosis are closely associated with formation of Aβ plaques and tau and contributes to inflammation and neurotoxicity and as well have beneficial functions in an appropriate process. Astro-degeneration may directly contribute to astroglial dysfunction of homeostatic support in the NVU and tripartite synapses, leading to a chronic NVU disruption, BBB breakdown and synaptic dysfunction. Further studies should focus on the underlying mechanism of multifaceted functions of astrocytes and novel therapeutic strategies for AD.

## Author Contributions

C-YL, YY and W-NJ searched the literature and drafted the manuscript. XW and H-LZ critically revised the manuscript. All authors listed have made a substantial, direct and intellectual contribution to the work, and approved it for publication.

## Conflict of Interest Statement

The authors declare that the research was conducted in the absence of any commercial or financial relationships that could be construed as a potential conflict of interest.

## Abbreviations

3xTg-AD: triple transgenic animal model of Alzheimer’s disease
AA: arachidonic acid
Aβ: amyloid beta
AD: Alzheimer’s disease
ALS: amyotrophic lateral sclerosis
AQP4: aquaporin 4
ATP: adenosine triphosphate
ATPase: adenosine triphosphatase
BBB: blood brain barrier
BDNF: brain-derived neurotrophic factor
C1q: complement component 1, q subcomponent
Ca^2+^: calcium
CBF: cerebral blood flow
CCL: chemokine C-C motif ligand
CNS: central nervous system
CNTF: cilliary neurotrophic factor
CSF: cerebrospinal fluid
CXCL: chemokine C-X-C motif ligand
EC: entorhinal cortex
EET: epoxyeicosatrienoic acid
FGF: fibroblast growth factor
GABA: γ-aminobutyric acid
G-CSF: granulocyte colony stimulating factors
GFAP: glial fibrillary acidic protein
GLT: glutamate transporter
GM-CSF: granulocyte-macrophage colony stimulating factors
GS: glutamine synthetase
HD: Huntington’s disease
IGFR: insulin-like growth factor receptor
IL: interleukin
INF: interferon
IP-10: INF-γ-induced protein 10
ISF: interstitial fluid
JAK: Janus kinase
K^+^: potassium
LIF: leukemia inhibitory factor
LPS: lipopolysaccharide
LTP: long-term potentiation
MCP-1: monocyte chemoattractant protein 1
MIF: macrophage migration inhibitory factor
MIP-1α: macrophage inflammatory protein 1 alpha
MS: multiple sclerosis
NFT: neurofibrillary tangle
NO: nitric oxide
NVU: neuro-vascular unit
PD: Parkinson’s disease
PH: potential of hydrogen
PGE2: prostaglandin E2
ROS: reactive oxygen species
STAT3: signal transducer and activator of transcription 3
TGF: transforming growth factor
TNF: tumor necrosis factor
ZO-1: zonula occludens-1.
